# Effect of serum albumin, a component of human pleural fluid, on transcriptional and phenotypic changes on *Acinetobacter baumannii* A118

**DOI:** 10.1007/s00284-021-02649-9

**Published:** 2021-09-14

**Authors:** Casin Le, Camila Pimentel, Marisel R. Tuttobene, Tomas Subils, Krisztina M. Papp-Wallace, Robert A. Bonomo, Luis A. Actis, Marcelo E. Tolmasky, Maria Soledad Ramirez

**Affiliations:** 1 Center for Applied Biotechnology Studies, Department of Biological Science, College of Natural Sciences and Mathematics, California State University Fullerton, Fullerton, California, USA; 2 Área Biología Molecular, Facultad de Ciencias Bioquímicas y Farmacéuticas, Universidad Nacional de Rosario, Rosario, Argentina; 3 Instituto de Procesos Biotecnológicos y Químicos de Rosario (IPROBYQ, CONICET-UNR), Rosario, Argentina; 4 Research Service, Veterans Affairs Northeast Ohio Healthcare System, Cleveland, Ohio, USA; 5 Departments of Medicine, Biochemistry, Proteomics and Bioinformatics, Case Western Reserve University School of Medicine, Cleveland, Ohio, USA; 6 CWRU-Cleveland VAMC Center for Antimicrobial Resistance and Epidemiology (Case VA CARES), Cleveland, Ohio, USA; 7 GRECC, Veterans Affairs Northeast Ohio Healthcare System, Cleveland, Ohio, USA; 8 Departments of Pharmacology, Molecular Biology and Microbiology, Case Western Reserve University School of Medicine, Cleveland, Ohio, USA; 9 Department of Microbiology, Miami University, Oxford, Ohio 45056, USA

**Keywords:** *Acinetobacter baumannii*, human serum albumin, human pleural fluid, quorum sensing, biofilm formation

## Abstract

*Acinetobacter baumannii* is a multidrug resistant pathogen that causes numerous infections associated with high mortality rates. Exposure to human body fluids, such as human pleural fluid (HPF) and human serum, modulates gene expression in *A. baumannii*, leading to changes in its pathogenic behavior. Diverse degrees of effects at the transcriptional level were observed in susceptible and carbapenem-resistant strains. The transcriptional analysis of AB5075, a hyper-virulent and extensively drug-resistant strain showed changes in genes associated with quorum sensing, quorum quenching, fatty acids metabolism, and high-efficient iron uptake systems. In addition, the distinctive role of human serum albumin (HSA) as a critical component of HPF was evidenced. In the present work, we used model strain to analyze more deeply into the contribution of HSA in triggering *A. baumannii’s* response. By qRT-PCR analysis, changes in the expression level of genes associated with quorum sensing, biofilm formation, and phenylacetic acid pathway were observed. Phenotypic approaches confirmed the transcriptional response. HSA, a predominant component of HPF, can modulate the expression and behavior of genes not only in a hyper-virulent and extensively drug-resistant *A. baumannii* model, but also in other strains with a different degree of susceptibility and pathogenicity.

## Introduction

*Acinetobacter baumannii* accounts for high mortality rates in hospitalized patients. Capable of persisting under desiccation, nutrient starvation, and exposure to high concentrations of antimicrobial agents, *A. baumannii* prevails in diverse clinical settings and within the human host. Human pleural fluid (HPF) was recently shown to alter the phenotypic behavior and expression of genes in *A. baumannii* (1). The effect was observed in an antibiotic-susceptible model strain *A. baumannii* A118, and in a hypervirulent and multi-drug resistant representative strain *A. baumannii* AB5075 (2). In the AB5075 strain, the expression of genes associated with quorum sensing, quorum quenching, fatty acid metabolism, and high-efficient iron uptake systems was affected by HPF; moreover, the distinctive role of human serum albumin (HSA) as a central component of HPF was found to be involved in AB5075’s response (2). To further understand the specific contribution of HSA as an important component of HPF in triggering *A. baumannii’s* response, we studied its effects on the antibiotic-susceptible model A118 strain.

## Materials and Methods

### Bacterial Strains

*Acinetobacter baumannii* strain A118 was used as bacterial models. This strain has been shown to be susceptible to a variety of antibiotics (3, 4).

### HSA Depletion

To obtain HSA-depleted HPF (dHPF), 1 mL of HPF was placed into a 3 kDa Amicon™ Ultra Centrifugal Filter (Millipore, Temecula, CA, USA) and the solution was centrifuged at 20,000 *g* for 10 min, as previously described (2). SDS-PAGE was carried out to confirm HSA depletion as previously stated (2).

### N-Acyl Homoserine Lactone (AHL) Detection

*Agrobacterium tumefaciens* NT1 (pZLR4) was used to detect the presence of AHLs in *A. baumannii* cultures as previously described (5, 6). The *A. tumefaciens* NT1 (pZLR4) AHL biosensor, which contains a plasmid-localized traG-lacZ fusion (pZLR4) responds to AHLs of chain lengths ranging from C6 to C12 (7). Briefly, 500 μL of the homogenate were loaded in a central well of 0.7% LB agar plates supplemented with 40 μg of 5-bromo-3- indolyl-b-D-galactopyranoside (X-Gal) per mL and 250 μL (OD = 2.5) of overnight cultures of *A. tumefaciens* biosensor. The presence of AHL was determined by the development of the blue color. As a positive control, 100 μL of N-Decanoyl-DL-homoserine lactone (C10-AHL) 12.5 mg/mL was utilized. Quantification of 5,5’-dibromo-4,4’-dichloro-indigo production in different conditions was determined by measuring the intensity of each complete plate and subtracting the intensity measured in the negative control, using ImageJ software (NIH). The values were normalized to the positive control, which received the arbitrary value of 100. Experiments were performed in triplicate. Significance differences were determined by ANOVA followed by Tukey’s multiple comparison test (*p* < 0.05), using GraphPad Prism (GraphPad Software, San Diego, CA, USA).

### Biofilm Assays

Biofilms assays were performed as previously described (2, 8). A118 cells were cultured in LB or LB supplemented with 4% HPF, 4% dHPF, or 4% dHPF + 0.2% HSA with agitation for 18 h at 37 °C. Following, the optical density at 600 nm (OD600) of each culture was adjusted to 0.9–1.1, vortexed, and diluted 1:100 in LB broth before being plated in technical triplicate in a 96-well polystyrene microtiter plate and being incubated at 37 °C for 24 h without agitation. The following day, the OD600 (ODG) was measured using a microplate reader (SpectraMax M3 microplate/ cuvette reader with SoftMax Pro v6 software) to determine the total biomass. Wells were emptied with a vacuum pipette, washed three times with 1X phosphate-buffered saline (PBS), and stained with 1% crystal violet (CV) for 15 m. Excess CV was removed by washing three more with 1X PBS and the biofilm-associated with the CV was solubilized in ethanol acetate (80:20) for 30 m. The OD580 (ODB) was measured using a microplate reader and results were reported as the ratio of biofilm to total biomass (ODB/ODG). Experiments were performed in triplicate, statistical analysis (Mann-Whitney test) was performed using GraphPad Prism (GraphPad Software, San Diego, CA, USA), and a *p* < 0.05 was considered significant.

### RNA Extraction and Quantitative Reverse Transcription Polymerase Chain Reaction (qRT-PCR)

RNA extracted and DNase-treated from *A. baumannii* strain A118 grown in LB and LB supplemented with 4% HPF, 4% dHPF, or 4% dHPF + 0.2% HSA, was used to synthesized cDNA using the manufacturer protocol provided within the iScriptTM Reverse Transcription Supermix for qPCR (Bio-Rad, Hercules, CA, USA). The cDNA concentrations were adjusted to a concentration of 50 ng/μL. qPCR was conducted using the iQTM SYBR®Green Supermix through the manufacturer’s instructions. At least three biological replicates of cDNA were used and were run in quadruplets. All samples were then run on the CFX96 TouchTM Real-Time PCR Detection System (Bio-Rad, Hercules, CA, USA). The transcript levels of each sample were normalized to the rpoB transcript levels for each cDNA sample. The relative quantification of gene expression was performed using the comparative threshold method 2^-ΔΔCt^. The ratios obtained after normalization were expressed as folds of change compared with cDNA samples isolated from bacteria cultures on LB. Significance differences were determined by ANOVA followed by Tukey’s multiple comparison test (*p* < 0.05), using GraphPad Prism (GraphPad Software, San Diego, CA, USA).

## Results and Discussion

**Figure 1.**
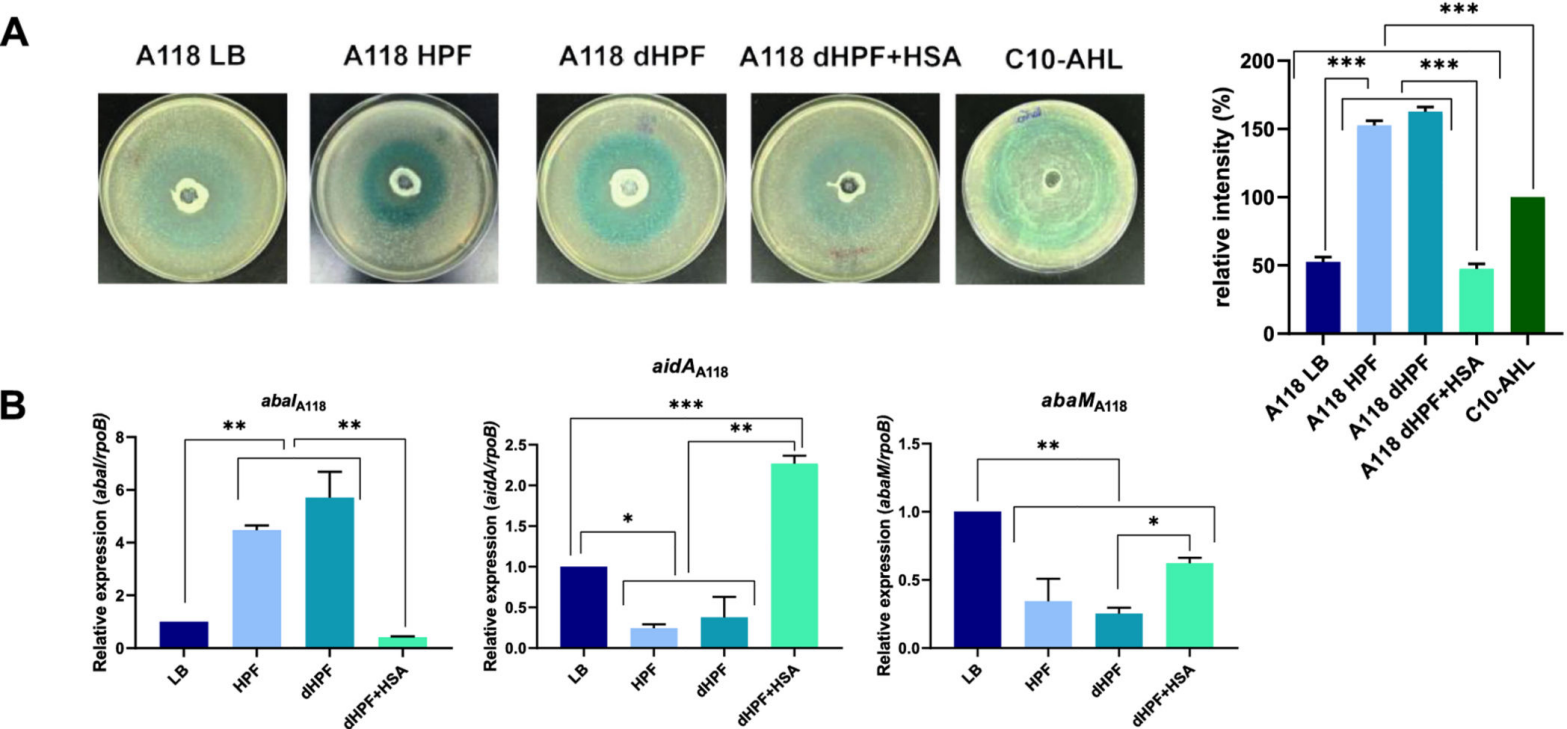
Phenotypic and genetic analysis of quorum sensing coding genes. A) N-Acyl Homoserine Lactone (AHL) detection in *A. baumannii* A118 supernatants of bacteria cultured in LB or LB supplemented with HPF, dHPF or dHPF + HSA using *Agrobacterium tumefaciens* NT1 pZLR4 biosensor strain. The presence of AHLs was revealed by the development of a blue color. Quantification of 5,5’-dibromo-4,4’-dichloro-indigo was estimated as the percentage relative to the C10-AHL standard, measured with ImageJ (NIH). B) qRT-PCR of A118 strain of the *abaI, aidA* and *abaM* genes associated with quorum sensing expressed in LB or LB supplemented with HPF, dHPF, or dHPF + HSA. Fold changes were calculated using double ΔCt analysis. At least three independent samples were used. LB was used as the reference condition. The mean ± SD is informed. Statistical significance (*p* < 0.05) was determined by ANOVA followed by Tukey’s multiple-comparison test, one asterisks: *p* < 0.05; two asterisks: *p* < 0.01 and three asterisks: *p* < 0.001.

Quorum sensing was identified as one of the mechanisms modified in *A. baumannii* AB5075 when HSA was present in the growth medium. To further confirm this response in an unrelated A118 strain, the impact of HSA on quorum sensing, assessed via N-acyl-homoserine lactone (AHL) production, was determined using *Agrobacterium tumefaciens*-based solid plate assays (6). Figure 1A shows that the supernatant recovered from the A118 strain cultured in LB broth produced a blue halo, indicating the presence of AHLs. In addition, when cultured in the presence of HPF or dHPF (HPF depleted of HSA), *A. baumannii* A118 produced darker blue halos indicative of higher amounts of AHLs. On the other hand, when cultured in the presence of dHPF supplemented with HSA, strain A118 produced a low-intensity blue halo (Fig. 1A). HSA induced degradation or inhibition of synthesis of large chain AHLs in *A. baumannii* A118, an effect that was also observed with the AB5075 strain (2).

To evaluate this response at the transcriptional level, we carried out qRT-PCR assays using total RNA extracted from *A. baumannii* A118 cells cultured in LB or LB supplemented with HPF, HSA-depleted HPF (dHPF), or dHPF supplemented with HSA (dHPF + HSA) (Figure 1 B). The results of these experiments revealed that the level of expression of *abaI*, which codes for AHLs synthase (9), was higher in cultures containing HPF and dHPF (4.46- and 5.7-fold increase, respectively), while in presence of dHPF + HSA the expression of *abaI* was decreased (0.417-fold) (Fig. 1 B). The qRT-PCR results further demonstrated that the expression levels of *aidA,* which is an *A. baumannii* lactonase responsible for AHL degradation (10), were 0.24- and 0.37- fold decreased when grown under HPF and dHPF conditions, respectively. On the other hand, *A. baumannii* A118 cultured in the dHPF + HSA condition produced a 2.86-fold increase in expression of *aidA*. The *abaM* gene, which encodes a negative regulator of quorum sensing, AbaM (9), was down-regulated in all culture conditions respect LB (0.34-fold, 0.25-fold and 0.62-fold decrease under HPF, dHPF and dHPF + HSA treatments, respectively) (Fig. 1B).

These results suggest that HSA induces changes in the expression of genes associated with quorum sensing, leading to a lower synthesis and/or increased degradation of AHLs. The intense blue color observed with the addition of HPF may be the consequence of reactive oxygen species (ROS) present in this fluid that induces higher production of AHLs.

**Figure 2.**
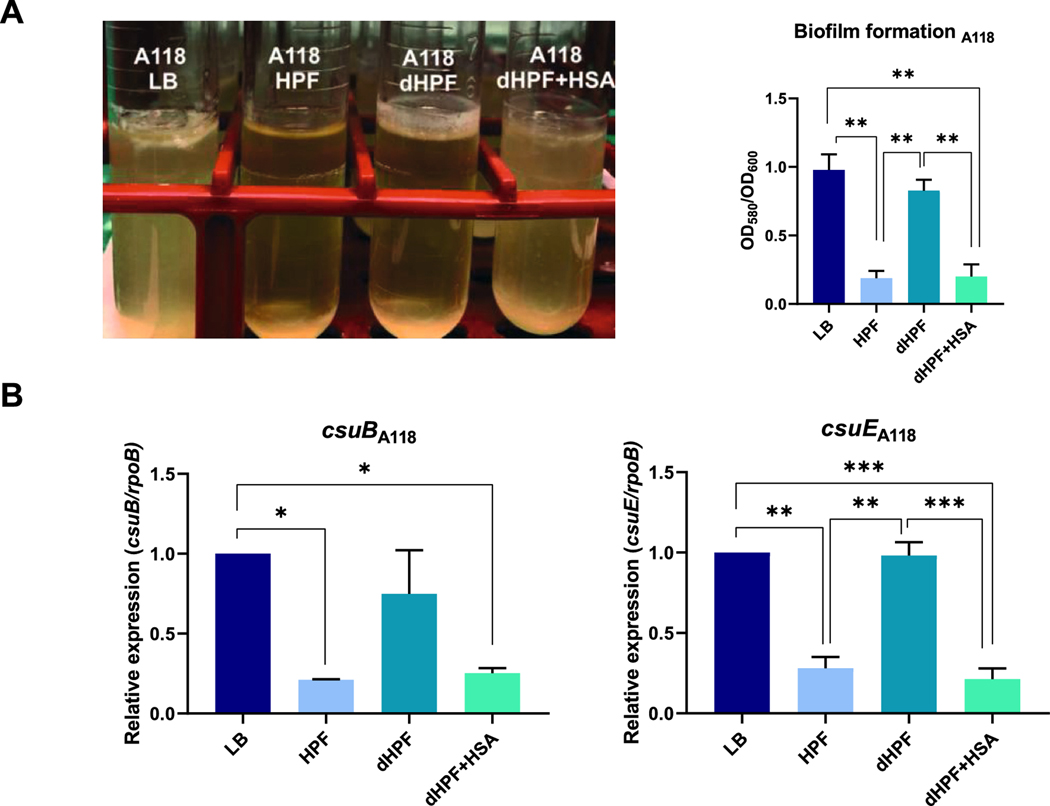
Phenotypic and genetic analysis of biofilm formation coding genes. A) Biofilm assays performed with the A118 strain grown in LB or LB supplemented with HPF, dHPF, or dHPF + HSA. B) qRT-PCR of the *csuB* and *csuE* biofilm formation-related genes in cells cultured in LB or LB supplemented with HPF, dHPF or dHPF + HSA. Fold changes were calculated using double ΔCt analysis. At least three independent samples were used. LB was used as the reference condition. The mean ± SD is informed. Statistical significance (*p* < 0.05) was determined by ANOVA followed by Tukey’s multiple-comparison test, one asterisks: *p* < 0.05; two asterisks: *p* < 0.01 and three asterisks: *p* < 0.001.

Consistent with the presumed role of HSA in biofilm formation, discernible differences occurred when *A. baumannii* A118 was cultured in a medium with different supplements (Fig. 2A). The presence of HSA, i.e., medium supplemented with HPF or dHPF + HSA, was associated with a less developed biofilm formation, while under LB or dHPF conditions a robust biofilm was observed (Fig. 2A). Also, two components of the *csu* operon, *csuB* and *csuE*, were downregulated in cells cultured in the presence of HPF or dHPF + HSA (Fig. 2B). This operon plays an important role in biofilm formation on abiotic surfaces in *A. baumannii* (11).

**Figure 3.**
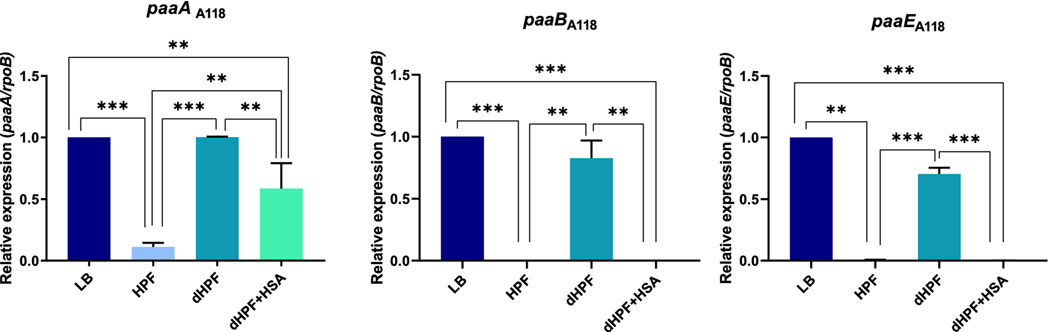
Genetic analysis of phenylacetic acid pathway related coding genes. qRT-PCR of *paaA, paaB* and *paaE,* genes related with the phenylacetic acid pathway, in A118 cells grown in LB or LB supplemented with HPF, dHPF or dHPF + HSA. Fold changes were calculated using double ΔCt analysis. At least three independent samples were used. LB was used as the reference condition. The mean ± SD is informed. Statistical significance (*p* < 0.05) was determined by ANOVA followed by Tukey’s multiple-comparison test, one asterisks: *p* < 0.05; two asterisks: *p* < 0.01 and three asterisks: *p* < 0.001.

Lastly, as the phenylacetic acid pathway (PA) contributes to host immune evasion and virulence, we analyzed the expression of representative genes of this pathway, *paaA*, *paaB* and *paaE* (12). We observed decreases in the expression levels of all *paa* genes analyzed in cells grown in media supplemented with HPF or dHPF + HSA (Fig.3).

## Conclusion

The results described herein support the role of HSA as one of the HPF components that affect the expression of genes related to quorum sensing, biofilm formation, and phenylacetic acid production, all features associated with *A. baumannii* pathobiology. A similar HSA modulatory response was previously observed with the AB5075 strain (2). Taken together, the results obtained with *A. baumannii* A118 and AB5075 suggest a general response of *A. baumannii* when grown in the presence of HSA. This data showed that *A. baumannii*’s ability to control its transcriptional and phenotypical response could be one of the factors contributing to *A. baumannii’s* adaptability to overcome stressful conditions within the host. Future work will be performed using a panel of different strains to observe if the modulatory response is ubiquitous, as well as, to identify the mechanism involved in trigerring *A. baumannii’s* response.
